# Substrate oxidation in primary human skeletal muscle cells is influenced by donor age

**DOI:** 10.1007/s00441-020-03275-w

**Published:** 2020-09-08

**Authors:** Vigdis Aas, G. Hege Thoresen, Arild C. Rustan, Jenny Lund

**Affiliations:** 1 Department of Life Sciences and Health, Faculty of Health Sciences, OsloMet - Oslo Metropolitan University, Oslo, Norway; 2 Section for Pharmacology and Pharmaceutical Biosciences, Department of Pharmacy, University of Oslo, Blindern, P.O. Box 1068, 0316 Oslo, Norway; 3 Department of Pharmacology, Institute of Clinical Medicine, University of Oslo, Oslo, Norway

**Keywords:** Skeletal muscle cells, Energy metabolism, Age, Body mass index (BMI), Muscle of origin, Gender

## Abstract

Primary human myotubes represent an alternative system to intact skeletal muscle for the study of human diseases related to changes in muscle energy metabolism. This work aimed to study if fatty acid and glucose metabolism in human myotubes in vitro were related to muscle of origin, donor gender, age, or body mass index (BMI). Myotubes from a total of 82 donors were established from three different skeletal muscles, i.e., *musculus vastus lateralis*, *musculus obliquus internus abdominis*, and *musculi interspinales*, and cellular energy metabolism was evaluated. Multiple linear regression analyses showed that donor age had a significant effect on glucose and oleic acid oxidation after correcting for gender, BMI, and muscle of origin. Donor BMI was the only significant contributor to cellular oleic acid uptake, whereas cellular glucose uptake did not rely on any of the variables examined. Despite the effect of age on substrate oxidation, cellular mRNA expression of pyruvate dehydrogenase kinase 4 (*PDK4*) and peroxisome proliferator–activated receptor gamma coactivator 1 alpha (*PPARGC1A*) did not correlate with donor age. In conclusion, donor age significantly impacts substrate oxidation in cultured human myotubes, whereas donor BMI affects cellular oleic acid uptake.

## Introduction

Primary human skeletal muscle cells (myotubes) derived from satellite cells represent an alternative system to intact skeletal muscle that can be used for the study of human diseases related to changes in energy metabolism (Aas et al. 2013; Henry et al. 1995). It has been observed that myotubes display morphological, metabolic, and biochemical similarities to adult skeletal muscle (Gaster et al. 2001; Henry et al. 1995; Thompson et al. 1996). This cell model has the most relevant genetic background, and because these cells are not immortalized, they allow investigation of the innate characteristics of the donors from whom the cells were obtained (Aas et al. 2013). In addition, the extracellular environment can be precisely controlled and modulated ex vivo making myotubes a valuable system for research.

Cultured human myotubes retain some of the phenotypic traits of their donors. For instance, the diabetic phenotype is conserved in myotubes established from subjects with obesity/type 2 diabetes as they exhibit primary defects in both glucose and lipid metabolism (Corpeleijn et al. 2010; Gaster et al. 2004; Kase et al. 2015). It has also been shown that myotubes established from athletic subjects have enhanced glucose and lipid metabolism compared with myotubes from sedentary subjects (Lund et al. 2018a, b). Moreover, the ability to switch between glucose and fatty acid oxidation (metabolic switching) is preserved in human myotubes, which supports the hypothesis that metabolic switching is an intrinsic property of skeletal muscle (Ukropcova et al. 2005). In addition to the genetic background, epigenetic mechanisms are probably involved in retaining the in vivo characteristics of the donors in the cultured cells (Aas et al. 2013; Lund et al. 2017).

Obesity, along with insulin resistance and type 2 diabetes mellitus, is linked to reduced lipid oxidation during fasting and impaired postprandial switch from lipid to glucose oxidation (Kelley et al. 1999). Furthermore, it has been observed that skeletal muscle cells from obese subjects have reduced capacity to oxidize fatty acids compared with myotubes from lean subjects, which is associated with lipid accumulation and insulin resistance (Holloway et al. 2009).

It is known that there are differences with regard to energy metabolism between genders in vivo (Wang et al. 2011). Women seem to predominantly oxidize carbohydrates in the postprandial state (Levadoux et al. 2001; Uranga et al. 2005), whereas for energy expenditure under higher energy demand, e.g., during exercise, fatty acid oxidation is the predominant source (Tarnopolsky 2008). Furthermore, skeletal muscles of women seem to be better prepared for storage and oxidation of lipids than male skeletal muscles, which in turn facilitate a higher turnover of intramyocellular triacylglycerols (Lundsgaard and Kiens 2014).

Another factor that affects several metabolic processes in skeletal muscle in vivo is age. With increasing age, it has been observed a decrease in insulin sensitivity (Johannsen et al. 2012; Lanza and Nair 2008; Ryan 2000), increased content of intramyocellular triacylglycerol (Crane et al. 2009), increased proportion of fast muscle fibers (D’Antona et al. 2003), reduced skeletal muscle mass (Lexell et al. 1988), reduced content of skeletal muscle peroxisome proliferator–activated receptor delta (Nilsson et al. 2007), reduced mitochondrial content (Crane et al. 2009; Rooyackers et al. 1996), and function (Johannsen et al. 2012). Increased age has also been associated with a decline in mitochondrial DNA, mRNA abundance, and mitochondrial ATP production (Hebert et al. 2010; Short et al. 2005; Welle et al. 2003). However, it has been suggested that a combination of obesity and inactivity, and not age per se, is most important in the age-related decline in insulin sensitivity (Lanza and Nair 2008).

An important aspect in evaluating research of myotubes obtained from satellite cells is the heterogeneity of muscle fibers and muscles of origin. Mammalian skeletal muscle fibers can be classified based on contractile rates, metabolism, and expression of isoforms of muscle-specific contractile proteins (Schiaffino and Reggiani 2011). Although other specialized functional fiber types exist, most human muscles consist of mixtures of three fiber types (Schiaffino and Reggiani 2011). It has been shown that myotubes co-express myosin heavy chain (MHC) isoforms regardless of the origin of satellite cells (Aas et al. 2013; Bonavaud et al. 2001), demonstrating that myotubes differ from donor muscle with respect to MHC expression. We have observed that differentiated myotubes contain both slow and fast fiber types (Aas et al. 2013; Lund et al. 2018b). However, the plasticity potential of skeletal muscle cells has also been well described (Schiaffino and Reggiani 2011). To our knowledge, it has not previously been studied whether the muscle of origin affects energy metabolism in myotube cultures.

The aim of the present work was to study if energy metabolism in skeletal muscle cells in vitro was related to muscle of origin, gender, age, or body mass index (BMI) of the cell donors. Myotubes were established from three different skeletal muscles, i.e., *musculus* (*m.*) *vastus lateralis*, *m. obliquus internus abdominis*, and *musculi* (*mm.*) *interspinales* of healthy donors, and energy metabolism in myotubes was examined. We have combined functional metabolic data from five different cohorts, thereby including data from 82 healthy donors.

## Methods

### Materials

Corning^®^ CellBIND^®^ tissue culture plates were from Corning (Schiphol-Rijk, the Netherlands). Nunc^™^ Cell Culture Treated Flasks with Filter Caps, Nunc^™^ 96-MicroWell^™^ plates, DMEM-Glutamax^™^ low glucose with sodium pyruvate, DPBS (without Mg^2+^ and Ca^2+^), FBS, Trypsin-EDTA, penicillin-streptomycin (10,000 IE/ml), amphotericin B, High-Capacity cDNA Reverse Transcription Kit, TaqMan reverse transcription kit reagents, MicroAmp^®^ Optical 96-well Reaction Plate, MicroAmp^®^ Optical Adhesive Film, Power SYBR^®^ Green PCR Master Mix, and primers for TaqMan PCR were from Thermo Fisher Scientific (Waltham, MA, USA). Ultroser G was from Pall (Cergy-Saint-Christophe, France) and insulin (Actrapid^®^ Penfill^®^ 100 IE/ml) from Novo Nordisk (Bagsvaerd, Denmark). SkBM-kit (SkGM) and BioWhittaker^®^ PBS were from Lonza (Walkersville, MD, USA). Trypan blue 0.4% solution, DMSO, l-glutamine, BSA (essentially fatty acid free), l-carnitine, d-glucose, oleic acid (18:1, n-9), and HEPES were from Sigma-Aldrich (St. Louis, MO, USA). RNeasy Mini Kit was from QIAGEN (Venlo, the Netherlands). d-[^14^C(U)]Glucose (2.9–5 mCi/mmol) and [1-^14^C]oleic acid (56.3 mCi/mmol) were from PerkinElmer NEN^®^ (Boston, MA, USA). OptiPhase Supermix, 96-well Isoplate^®^, Unifilter^®^-96 GF/B, and TopSeal^®^-A transparent film were from PerkinElmer (Shelton, CT, USA). Bio-Rad Protein Assay Dye Reagent Concentrate was from Bio-Rad (Copenhagen, Denmark).

### Culturing of human myotubes

Multinucleated human myotubes were established by activation and proliferation of satellite cells isolated from a small muscle biopsy (100–200 mg) of *m. obliquus internus abdominis*, *m. vastus lateralis*, or *mm. interspinales*. For the proliferation of myoblasts, DMEM-Glutamax^™^ (5.5 mM glucose) medium supplemented with FBS (2%), Ultroser G (2%), HEPES, penicillin/streptomycin, gentamicin, and amphotericin B was used. At approximately 80% confluence, the culture medium was changed to DMEM-Glutamax^™^ (5.5 mM glucose) supplemented with FBS (2%), insulin (25 pM), penicillin/streptomycin, gentamicin, and amphotericin B to initiate differentiation into multinucleated myotubes. The cells were allowed to differentiate for 7 days. During the culturing process, muscle cells were incubated in a humidified CO_2_ (5%) atmosphere at 37 °C, and the medium was changed every 2 to 3 days.

### Substrate oxidation assay

Skeletal muscle cells (7000 cells/well) were cultured in 96-well CellBIND^®^ microplates. d-[^14^C(U)]Glucose (0.5 μCi/ml, 100 μM, or 200 μM) or [1-^14^C]oleic acid (0.5 μCi/ml, 100 μM) was given during 4-h trapping as described previously (Wensaas et al. 2007). In brief, a 96-well UniFilter^®^ microplate (PerkinElmer, Shelton, CT, USA), activated for the capture of CO_2_ by the addition of 1 M NaOH, was mounted on top of the 96-well plate. After incubation, the cells were washed and harvested in 0.1 M NaOH. The ^14^CO_2_ trapped in the filter and cell-associated (CA) glucose was measured with a 2450 MicroBeta^2^ scintillation counter (PerkinElmer). The sum of ^14^CO_2_ and remaining CA radioactivity was considered an estimation of total cellular uptake of substrate: CO_2_ + CA. All data were related to total cell protein concentration measured in the cell lysates by the Bio-Rad protein assay.

### RNA isolation and analysis of gene expression by real-time qPCR

Skeletal muscle cells were cultured and differentiated before total RNA was isolated using the RNeasy Mini Kit according to the supplier’s protocol. RNA was reversely transcribed with a High-Capacity cDNA Reverse Transcription Kit and TaqMan Reverse Transcription Reagents using a PerkinElmer Thermal Cycler 9600. Primers were designed using Primer Express^®^ and qPCR was performed using an ABI PRISM 7000 Detection system (Thermo Fisher Scientific). Expression levels were normalized to the housekeeping gene acidic ribosomal phosphoprotein P0 (*RPLP0*). Glyceraldehyde-3-phosphate dehydrogenase (*GAPDH*) was also analyzed as a housekeeping gene; there were no differences between normalizing for *RPLP0* or *GAPDH*. The following forward and reverse primers were used at a concentration of 30 μM: *GAPDH* (acc. no.: NM002046), pyruvate dehydrogenase kinase 4 (*PDK4*, acc. no.: BC040239), peroxisome proliferator–activated receptor gamma coactivator 1 alpha (*PPARGC1A*, acc. no.: NM013261.3), and *RPLP0* (acc. no.: M17885).

### Presentation of data and statistics

**Fig. 1:**
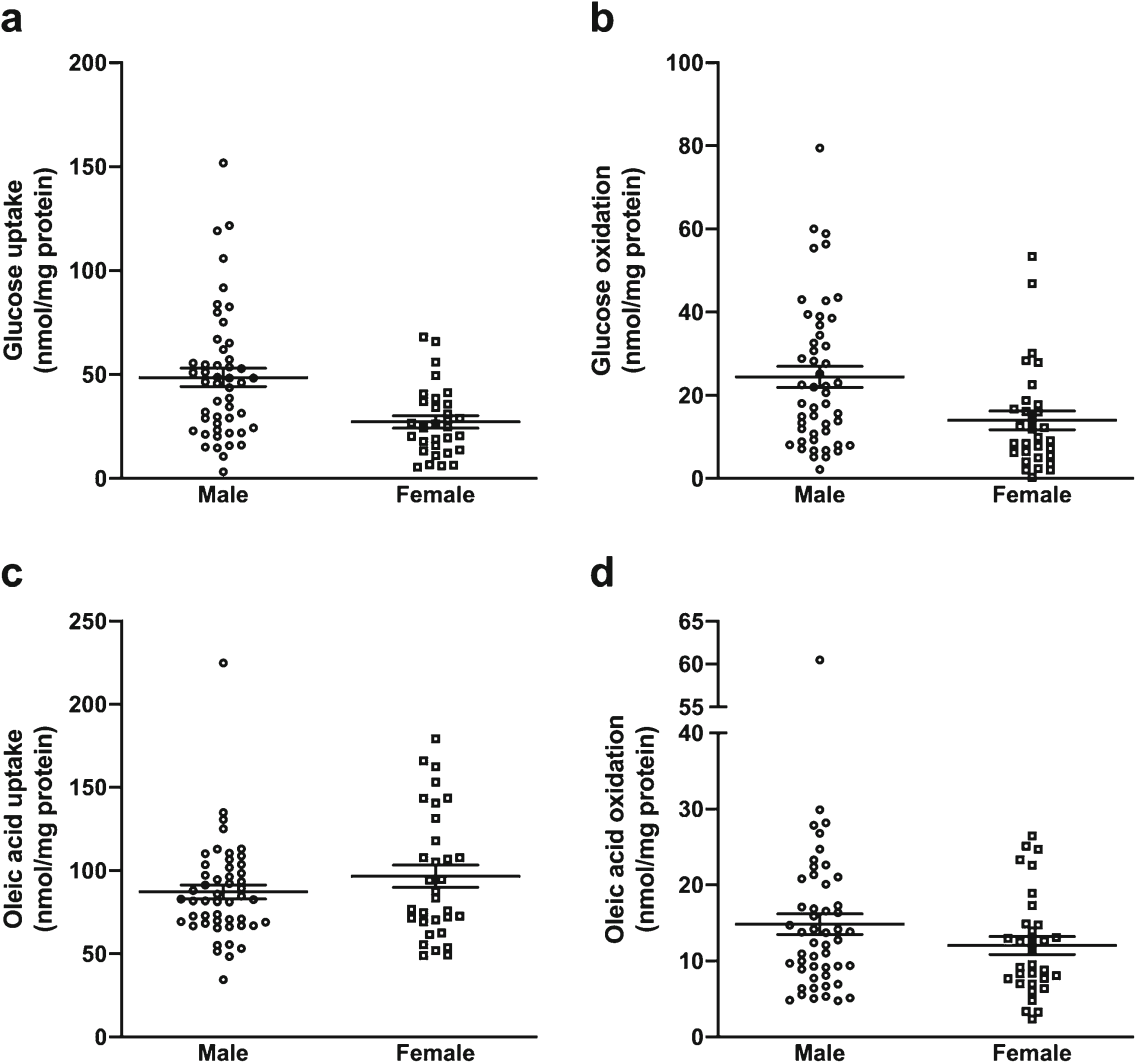
Cellular energy metabolism presented by gender. **a** Glucose uptake, **b** glucose oxidation, **c** oleic acid uptake, and **d** oleic acid oxidation in cultured myotubes established from male and female volunteers. Results are presented individually and with mean ± SEM from experiments on myotubes from **a**, **b** 80 (49 males and 31 females) participants or **c**, **d** 82 (50 males and 32 females) participants

**Fig. 2:**
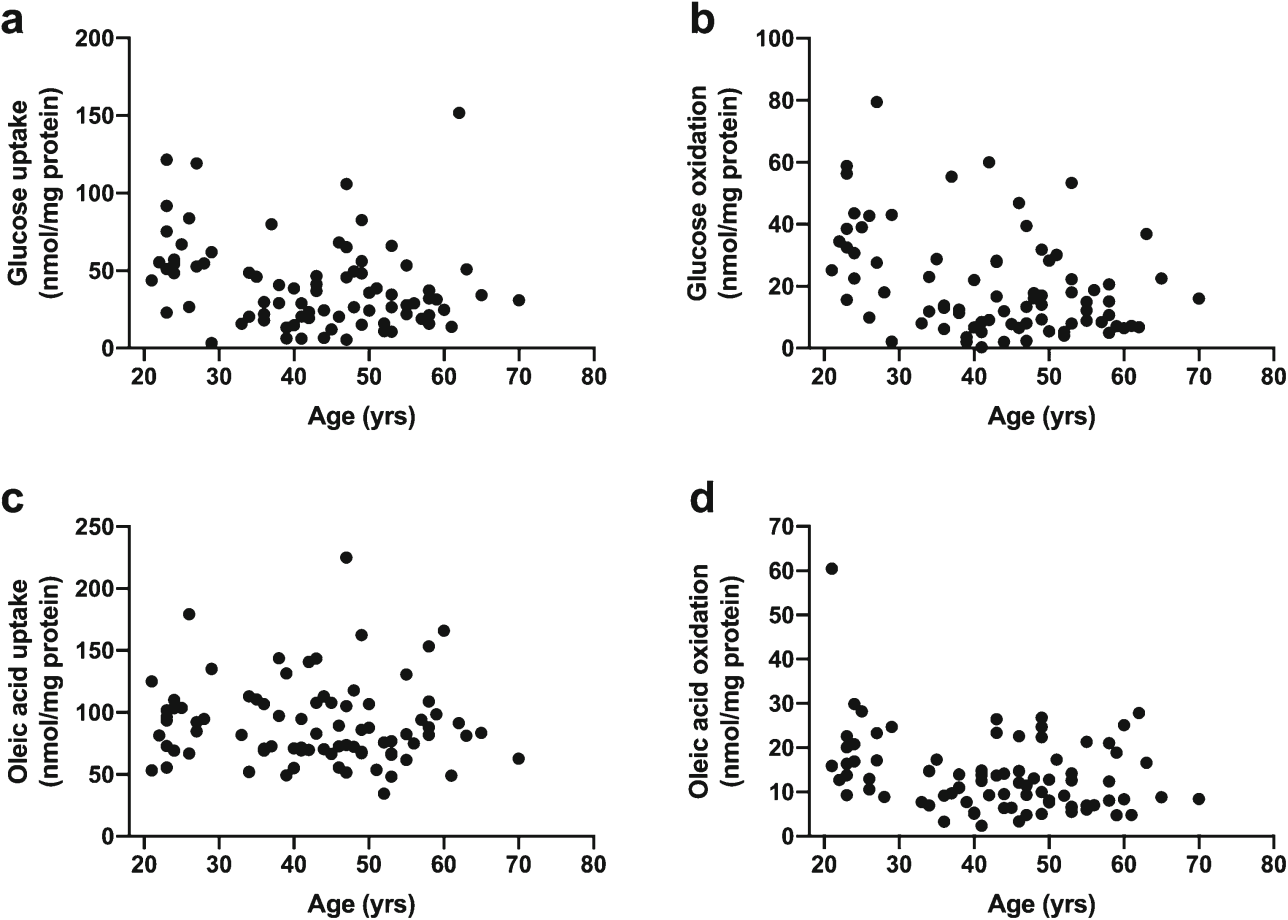
Cellular energy metabolism presented by donor age. **a** Glucose uptake, **b** glucose oxidation, **c** oleic acid uptake, and **d** oleic acid oxidation in cultured human myotubes. Results are presented individually from experiments on myotubes from **a**, **b** 80 or **c**, **d** 82 participants

**Fig. 3:**
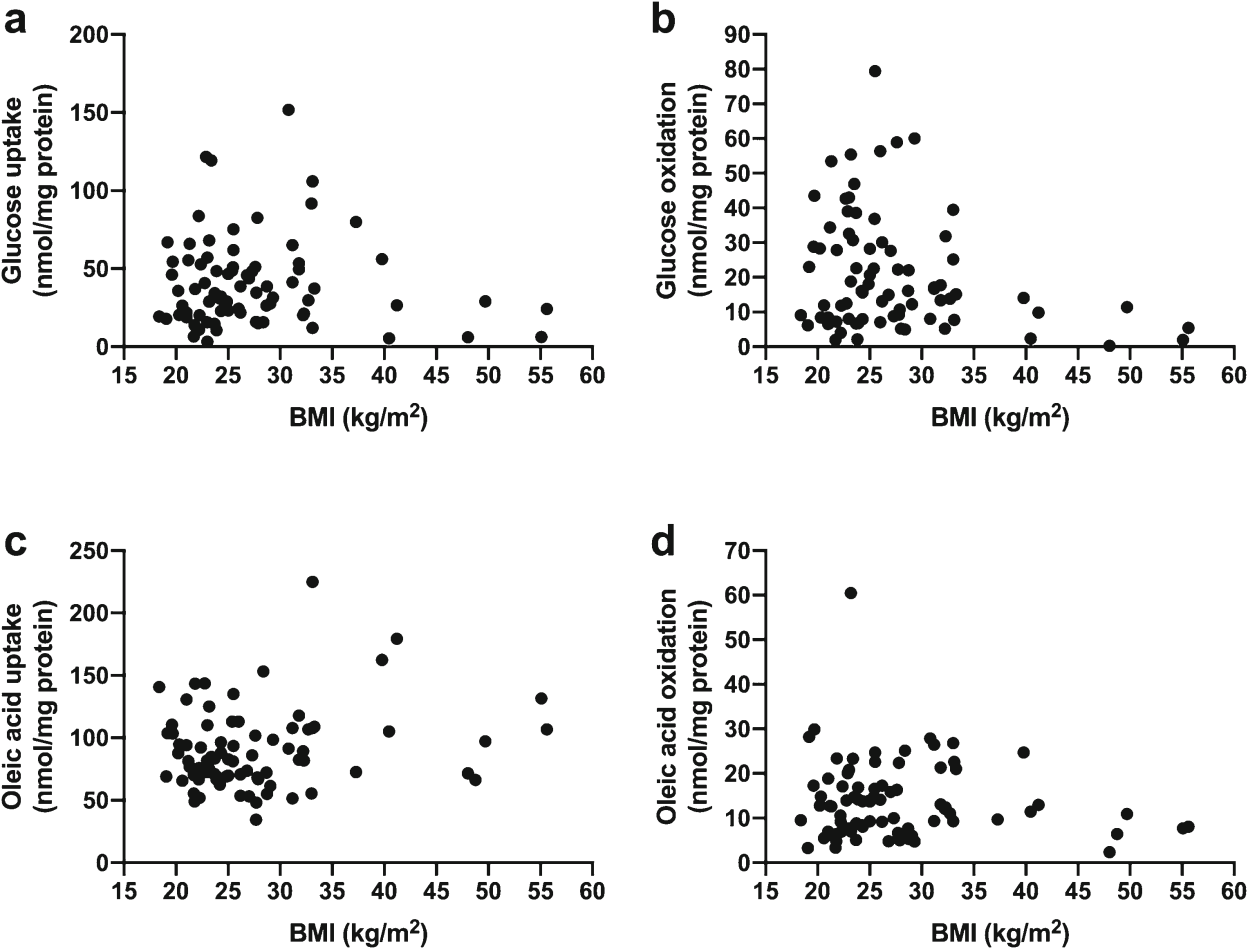
Cellular energy metabolism presented by body mass index (BMI) of the donors. **a** Glucose uptake, **b** glucose oxidation, **c** oleic acid uptake, and **d** oleic acid oxidation in cultured human myotubes. Results are presented individually from experiments on myotubes from **a**, **b** 80 or **c**, **d** 82 participants

**Fig. 4:**
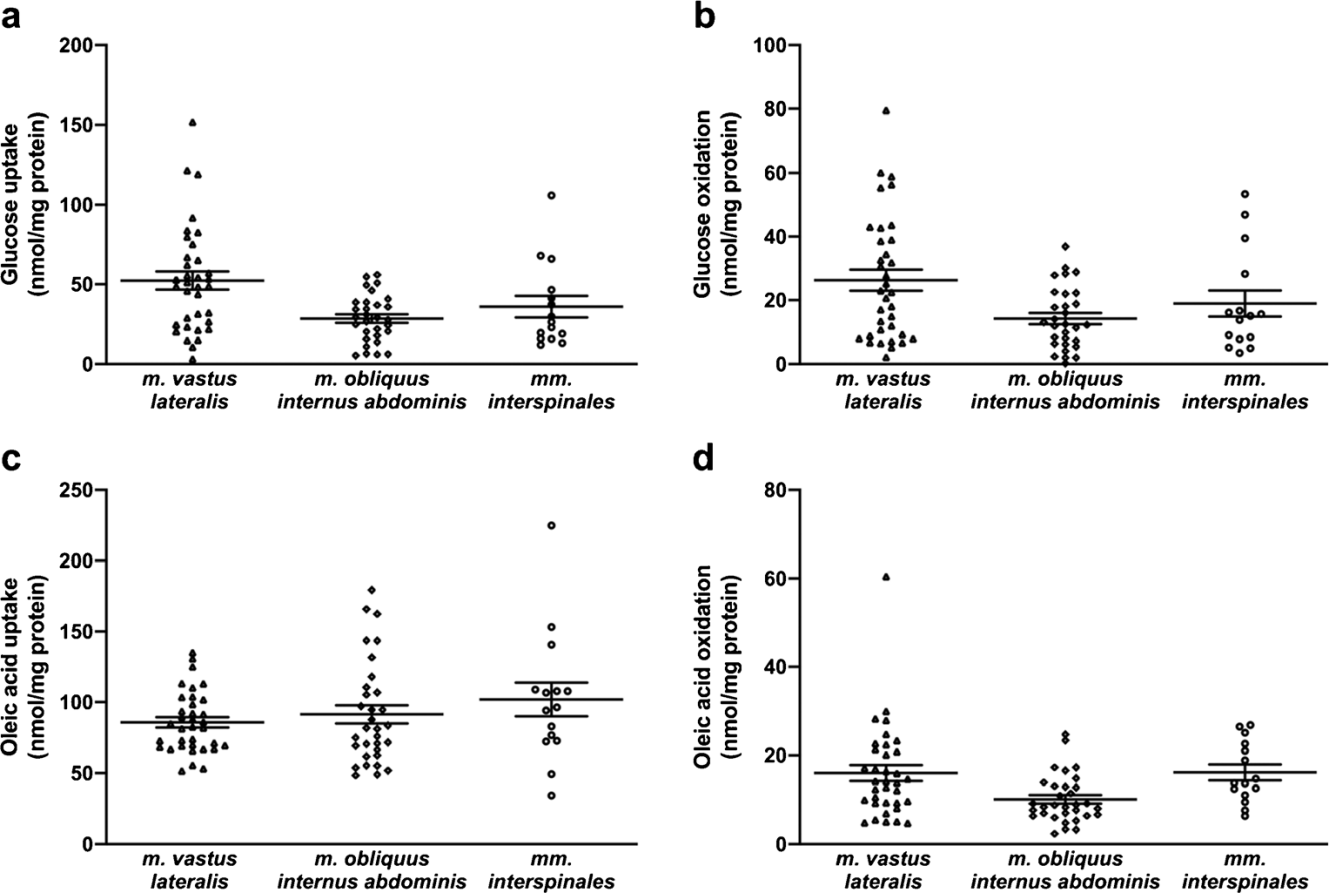
Cellular energy metabolism presented by the muscle of origin. **a** Glucose uptake, **b** glucose oxidation, **c** oleic acid uptake, and **d** oleic acid oxidation in cultured myotubes established from three different muscles. Results are presented individually and with mean ± SEM from 80 to 82 individual experiments: **a**, **b** 30 or **c**, **d** 32 experiments on myotubes from *m. obliquus internus abdominis*, 35 experiments on myotubes from *m. vastus lateralis*, and 15 experiments on myotubes from *mm. interspinales*

All individual data are presented in Figs. 1, 2, 3, and 4. The value *n* represents the number of different donors, each with at least duplicate observations. Statistical analyses were performed using GraphPad Prism 8 for Windows (GraphPad Software, Inc., La Jolla, CA, USA) or SPSS version 25 (IBM^®^ SPSS^®^ Statistics for Windows, Armonk, NY, USA). Two-way ANOVA with Tukey’s correction was used to evaluate differences between donors of different muscles (Table 1), whereas unpaired Student’s *t* test was used to evaluate differences between genders within a single muscle (Table 1). Multiple regression analyses were performed where age, BMI, gender, and muscle of origin (*m. obliquus internus abdominis* and *mm. interspinales* relative to *m. vastus lateralis*) were used as independent variables to determine substrate uptake and oxidation, whereas linear regression analyses were used to study the relationship between oleic acid oxidation and gene expressions of *PDK4* and *PPARGC1A* in myotubes. A *p* value ≤ 0.050 was considered significant.

**Table 1:** Donor characteristics

	*Musculus obliquus internus abdominis*		*Musculus vastus lateralis*		*Musculi interspinales*	
Gender	Female (*n* = 23)	Male (*n* = 9)	Female (*n* = 0)	Male (*n* = 35)	Female (*n* = 9)	Male (*n* = 6)
Age (years)	47.9 ± 2.2	41.2 ± 3.6	NA	38.0 ± 2.3	47.9 ± 2.2	43.2 ± 5.1
BMI (kg/m^2^)	29.6 ± 2.5	31.1 ± 4.1	NA	25.9 ± 0.7	25.7 ± 1.8	29.4 ± 1.7

Values are given as mean ± SEM. *BMI*, body mass index; *NA*, not available

## Results

In the current work, we have explored whether certain donor characteristics can affect metabolic findings in human myotubes. Energy metabolism in cultured cells was related to donor characteristics from five different myotube cohorts, established from satellite cells isolated from biopsies from three different skeletal muscles, from healthy male and female donors, with varying age and BMI (Table 1). By doing this, we have included data from up to 82 donors in total. There were no statistically significant differences in age or BMI between male and female donors among the different muscles, nor across different muscles. However, no biopsies were taken from *musculus vastus lateralis* of female donors, which is a weakness of the present study (Table 1).

### Cellular energy metabolism parameters presented by gender, donor age, body mass index, and muscle of origin

Our data set consists of 80 individual results from substrate oxidation experiments with radiolabeled glucose, and 82 individual results from substrate oxidation experiments with radiolabeled oleic acid. Individual data on glucose uptake, glucose oxidation, oleic acid uptake, and oleic acid oxidation in cultured myotubes are presented by gender (Fig. 1), donor age (Fig. 2), BMI (Fig. 3), and muscle of origin (Fig. 4).

### Multiple linear regression analyses

To explore the impact of donor age, gender, BMI, and muscle of origin on the metabolic parameters, and to avoid confounding factors when comparing variables of the cohorts, multiple regression analyses were performed for the statistical analyses. All independent variables (donor age, gender, BMI, and muscle of origin) were included in the model, and uptake and oxidation of glucose and oleic acid were used as dependent variables. To compare muscle of origin, *m. vastus lateralis* was used as reference muscle.

When controlling for BMI, gender, age, and muscle of origin, none of these in vivo variables had a significant effect on cellular glucose uptake, and only donor age had a significant impact on cellular glucose oxidation (Table 2).

**Table 2:** Multiple linear regression analysis between selected variables in vivo and glucose metabolism in cultured human myotubes

In vitro variable	In vivo variable	Beta	*p* value
Glucose uptake (model *R*^2^ = 0.177)	BMI	0.002	0.989
	Gender	0.124	0.432
	*mm. interspinales*	− 0.114	0.408
	Age	− 0.119	0.305
	*m. obliquus internus abdominis*	− 0.289	0.087
Glucose oxidation (model *R*^2^ = 0.207)	*mm. interspinales*	− 0.030	0.825
	Gender	0.081	0.601
	*m. obliquus internus abdominis*	− 0.167	0.310
	BMI	− 0.159	0.147
	Age	− 0.275	0.017*

*Statistically significant (*p* ≤ 0.050, multiple regression analysis) from 80 individual experiments (*n* = 80). *BMI*, body mass index; *m*., *musculus*; *mm*., *musculi*

The multiple regression analysis also showed that BMI was a statistically significant variable that affected oleic acid uptake and that donor age was the sole significant variable to impact cellular oleic acid oxidation (Table 3).

**Table 3:** Multiple linear regression analysis between selected variables in vivo and oleic acid metabolism in cultured human myotubes

In vitro variable	In vivo variable	Beta	*p* value
Oleic acid uptake (model *R*^2^ = 0.134)	*m. obliquus internus abdominis*	− 0.090	0.595
	*mm. interspinales*	0.178	0.201
	Gender	− 0.194	0.219
	Age	− 0.205	0.082
	BMI	0.224	0.050*
Oleic acid oxidation (model *R*^2^ = 0.174)	BMI	− 0.037	0.735
	*mm. interspinales*	0.047	0.729
	Gender	− 0.093	0.544
	*m. obliquus internus abdominis*	− 0.300	0.072
	Age	− 0.264	0.023*

*Statistically significant (*p* ≤ 0.050, multiple regression analysis) from 82 individual experiments (*n* = 82). *BMI*, body mass index; *m*., *musculus*; *mm*., *musculi*

Taken together, with advancing donor age, both glucose and oleic acid oxidation were reduced in cultured myotubes, whereas an increase in donor BMI resulted in increased cellular uptake of oleic acid. Glucose uptake was not significantly affected by gender, age, or BMI of the donors and did not differ between cells from different skeletal muscles.

### Correlations between gene expression of *PDK4* and *PPARGC1A* and donor age

**Fig. 5:**
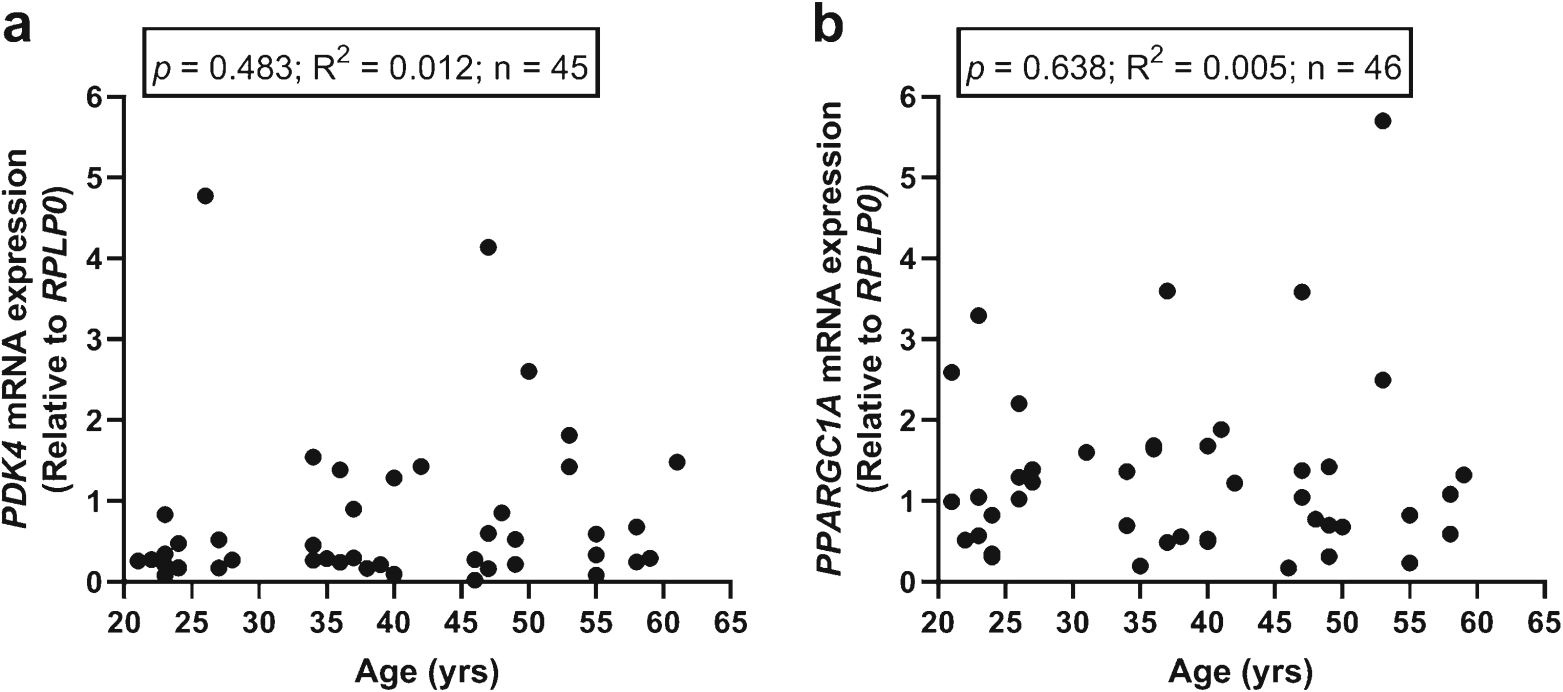
Impact of donor age on cellular mRNA expression of *PDK4* and *PPARGC1A*. Linear regression analysis between donor age and **a** cellular mRNA expression of pyruvate dehydrogenase kinase 4 (*PDK4*) relative to housekeeping gene ribosomal protein lateral stalk subunit P0 (*RPLP0*) and **b** cellular mRNA expression of peroxisome proliferator–activated receptor gamma coactivator 1 alpha (*PPARGC1A*) relative to housekeeping gene ribosomal protein lateral stalk subunit P0 (*RPLP0*)

PDK4 and PGC1α are two drivers of substrate oxidation, and given the impact age had on substrate oxidation in cultured human myotubes, we performed linear regression analyses between donor age and mRNA expression of *PDK4* and *PPARGC1A* in myotubes from a subset of the donors (45 and 46 donors, Fig. 5 a and b, respectively). The mRNA expression in the myotubes of these genes did not correlate with the age of the donors (Fig. 5).

## Discussion

In the present study, we have examined which in vivo variables that influence cellular uptake and oxidation of glucose and oleic acid in vitro in myotubes based on metabolic data from five different cohorts, including up to 82 donors. To our knowledge, no previous studies have included a sample size as large as the present study, with all cell experiments performed under the same experimental conditions. Only donor age was a statistically significant predictor for cellular substrate oxidation, whereas cellular uptake of oleic acid was dependent on the BMI of the donors. There were no statistically significant in vivo predictors for cellular glucose uptake.

Aging has been associated with reduced skeletal muscle function (Power et al. 2013) and mitochondrial dysfunction (Short et al. 2005). During the aging process, it has been observed increased mutations in mitochondrial DNA, decreased activity of certain mitochondrial enzymes, and reduced mitochondrial content with increased morphological changes (Peterson et al. 2012). Part of the decline in mitochondrial function appears to be due to alterations in mitochondrial synthesis and degradation, whereas the remainder is attributed to reduced physical activity (Carter et al. 2015). Given the central role of mitochondria in energy homeostasis, it was expected that myotube energy metabolism also would be affected by aging. In a study comparing myotubes from young and middle-aged men, it was shown a tendency towards lower glucose uptake in myotubes from aged men (Bunprajun et al. 2013). We observed a negative association between cellular glucose uptake and oxidation as well as oleic acid oxidation with donor age after correcting for gender, BMI, and muscle of origin.

Given the impact donor age had on substrate oxidation in the myotubes, we examined in a subset of the donors whether cellular mRNA expression of two important drivers of substrate oxidation, *PDK4* and *PPARGC1A*, correlated with donor age. PDK4 phosphorylates pyruvate dehydrogenase alpha 1 (PDHA1) at Ser300 and inhibits PDHA1 enzymatic activity, one of the enzymes necessary for driving glucose oxidation via mobilization of pyruvate for the tricarboxylic acid cycle. In our study, cellular mRNA expression of neither *PPARGC1A* nor *PDK4* correlated with donor age. In accordance with our results, in skeletal muscle biopsies, Consitt et al. did not observe different protein expression of PDK4 between younger and older subjects (Consitt et al. 2016) and Short et al. reported that mRNA expression of *PPARGC1A* in human skeletal muscle biopsies was unaffected by age (Short et al. 2003). However, several studies have reported that mRNA or protein expression of PGC1α is reduced in both slow- and fast-twitch skeletal muscle with increasing age (Chabi et al. 2008; Conley et al. 2007; Ghosh et al. 2011; Koltai et al. 2012; Short et al. 2005), suggesting that reductions in mitochondrial function or content may be put in association with loss of this important coactivator (Carter et al. 2015). Possible reasons for the different findings might be species differences, material examined, number of individual samples included, or whether it is mRNA or protein level that has been examined. Most of the former human studies have examined muscle biopsies (Ghosh et al. 2011), whereas we have used cultured myotubes. Furthermore, animal studies are a source of several of the other available data (Chabi et al. 2008; Koltai et al. 2012). Metabolism between murine animals and humans is different in many ways, particularly are mice and rats much more metabolically active than humans (Kleinert et al. 2018). Moreover, muscle cells in the present study were grown in the presence of glucose as the main energy source. If the myotubes had been treated with for instance fatty acids prior to examination of mRNA expressions, we might have been able to induce differences as these genes mainly are associated with lipid metabolism.

Most subjects with type 2 diabetes are overweight or obese (Smyth and Heron 2006). A high BMI is strongly associated with insulin resistance and type 2 diabetes (Alberti et al. 2007; James 2004), and weight loss is thus an important lifestyle change for prevention and treatment of these conditions (Tuomilehto et al. 2001). An enhanced glucose metabolism but unchanged fatty acid metabolism has been reported in cultured human skeletal muscle cells after Roux-en-Y gastric bypass surgery (Nascimento et al. 2015). Others have reported that cultured myotubes established from obese subjects have reduced capacity to oxidize fatty acids compared with cells obtained from lean subjects (Berggren et al. 2008; Boyle et al. 2012; Gaster 2009; Gaster et al. 2004). However, in our study, multiple linear regression analysis revealed that only oleic acid uptake, not oleic acid oxidation or glucose metabolism in myotubes, was affected by donor BMI. We have also recently observed that uptake of palmitic acid was higher in cultured human myotubes from obese compared with myotubes from lean subjects, whereas oxidation of palmitic acid was unaffected (Løvsletten et al. 2020). Furthermore, Bonen et al. described an approximately 4-fold higher transport rate of long-chain fatty acids and triacylglycerol content in giant sarcolemmal vesicles prepared from skeletal muscle biopsies from obese subjects (Bonen et al. 2004).

There have not, as far as we know, been performed any studies to compare energy metabolism in myotubes derived from satellite cells isolated from different human skeletal muscles. Our data set is based on myotubes derived from satellite cells from the three different skeletal muscles, i.e. *m. vastus lateralis*, *m. obliquus internus abdominis*, and *mm. interspinales*. Of the three muscles, the latter has, as far as we are aware of, not previously been used for skeletal muscle cellular metabolic studies. Murine and primate studies have shown that *mm. interspinales* contain a high amount of the slow, oxidative type I fibers, where the amount increased down the vertebras of the spine as the muscle size increased (Neufuss et al. 2014; Schilling 2009). In Fig. 4, there appeared to be slight differences between muscle types, but these were due to confounding factors, such as age.

Differences in skeletal muscle substrate metabolism between genders are well described in vivo (reviewed in Lundsgaard and Kiens (2014)). However, in cultured human myotubes, the gender difference does not seem to be maintained when examined under basal conditions (Rune et al. 2009; Salehzadeh et al. 2011). Studies have shown that the metabolism of glucose and palmitic acid was similar in myotubes from male and female donors (Rune et al. 2009). Despite mRNA expression of several genes important for the metabolism of glucose and fatty acids has been reported to be higher in skeletal muscle biopsies from women than men, the expression was similar in cultured myotubes from the same donors (Rune et al. 2009; Salehzadeh et al. 2011). In the current study, we did not find gender to be a significant determinant of myotube energy metabolism. However, a weakness with the present study is that we did not have cells from biopsies from *musculus vastus lateralis* of female donors.

To conclude, donor age had a significant impact on both glucose and oleic acid oxidation in cultured human myotubes, whereas BMI of the donors influenced only oleic acid uptake by the cells. Therefore, in studies aiming to study metabolic processes in cultured myotubes, the age of the muscle biopsy donors should be especially emphasized.
